# From polygenic risk to functional genomics: a framework for precision gynecological disease modeling

**DOI:** 10.1038/s41467-026-77342-1

**Published:** 2026-08-27

**Authors:** Masuma Khatun, Juha S. Tapanainen, Andres Salumets

**Affiliations:** 1 Department of Obstetrics and Gynecology, University of Helsinki and Helsinki University Hospital, Helsinki, Finland; 2 Department of Obstetrics and Gynecology, Institute of Clinical Medicine, University of Tartu, Tartu, Estonia; 3 Celvia CC, Tartu, Estonia; 4 Division of Obstetrics and Gynecology, Department of Clinical Science, Intervention and Technology, Karolinska Institute and Karolinska University Hospital, Stockholm, Sweden

**Keywords:** Endocrine reproductive disorders, Gynaecological cancer, Induced pluripotent stem cells

## Abstract

Gynecological conditions including endometriosis, polyendocrine metabolic ovarian syndrome and malignancies cause widespread chronic morbidity and infertility, yet translating genetic discoveries into biological mechanisms remains challenging. We propose a framework integrating biobank-derived human genetic data, patient-derived stem cell models, and advanced tissue engineering. By combining naturally occurring genetic variation with targeted genome engineering and high-resolution molecular analyses, this approach promises to enable mechanistic insights and therapeutic discovery. We discuss methodological, ethical, and translational challenges, including polygenic risk score construction, variant prioritization, ancestry-related limitations, and modeling hormonal microenvironments. Together, this framework offers a roadmap for advancing functional genomics and precision medicine across gynecological diseases.

## Abstract (web summary)

In this Perspective, the authors outline a framework integrating polygenic risk scores, induced pluripotent stem cells, genome engineering and advanced tissue models to bridge human genetics with precision gynecological disease research.

## Introduction

Gynecological diseases such as endometriosis, polyendocrine metabolic syndrome (PMOS; formerly known as polycystic ovary syndrome; PCOS), and reproductive tract malignancies impose a substantial global health burden and exhibit considerable heritability with complex genetic architectures^1^. Although genome-wide association studies (GWAS) have identified numerous susceptibility loci associated with these conditions^2,3^, translating these findings into biological interpretation remains challenging. Most risk variants reside within non-coding regions, where their functional effects are influenced by linkage disequilibrium, tissue-specific regulatory networks, and hormone-dependent biological processes. Consequently, many GWAS associations remain statistically robust, but mechanistically unresolved^4^.

Polygenic risk scores (PRS) aggregate the cumulative effects of common genetic variants into quantitative estimates of inherited susceptibility. While PRS have emerged as valuable tools for genetic risk prediction and stratification, they provide limited insight into the biological pathways through which genetic risk influences disease development^5^. Simultaneously, conventional in vitro models fail to capture the dynamic endocrine, immune, and stromal interactions that characterize reproductive tissues, whereas animal models are constrained by species-specific differences in reproductive physiology^6^. These shortcomings impede efforts to establish mechanistic links between genetic risk and disease-associated cellular phenotypes.

Induced pluripotent stem cells (iPSCs) offer a promising alternative by preserving donor-specific genetic backgrounds, while enabling differentiation into disease-relevant reproductive cell types. Patient-derived iPSC models can recapitulate key features of human reproductive biology and provide experimentally tractable platforms for studying genetically complex disorders^7,8^. Despite rapid advances in both stem cell biology and statistical genetics, the integration of polygenic risk information into iPSC-based disease modeling remains relatively underexplored.

To address this gap, we propose a conceptual framework that integrates PRS-informed donor stratification, iPSC-derived disease models, genome engineering, and advanced three-dimensional (3D) bioengineering platforms, including organoids and organ-on-chip systems. Rather than viewing PRS as mechanistic representations of disease biology, we position them as tools for identifying genetically informative donor populations, while iPSC-based models serve as experimental platforms for investigating how inherited genetic variation shapes cellular phenotypes and disease-relevant pathways. We also discuss key methodological considerations, including PRS construction, ancestry-specific performance, variant prioritization, and modeling of the hormonal microenvironment, together with the technical, ethical, and translational challenges that currently limit implementation. As a Perspective article, this work does not present experimental findings; instead, it outlines a testable framework and future research directions for linking population-scale genetic discoveries with functional genomics and stem cell-based models of gynecological disease.

## PRS-integrated iPSC modeling framework and donor stratification

**Fig. 1:**
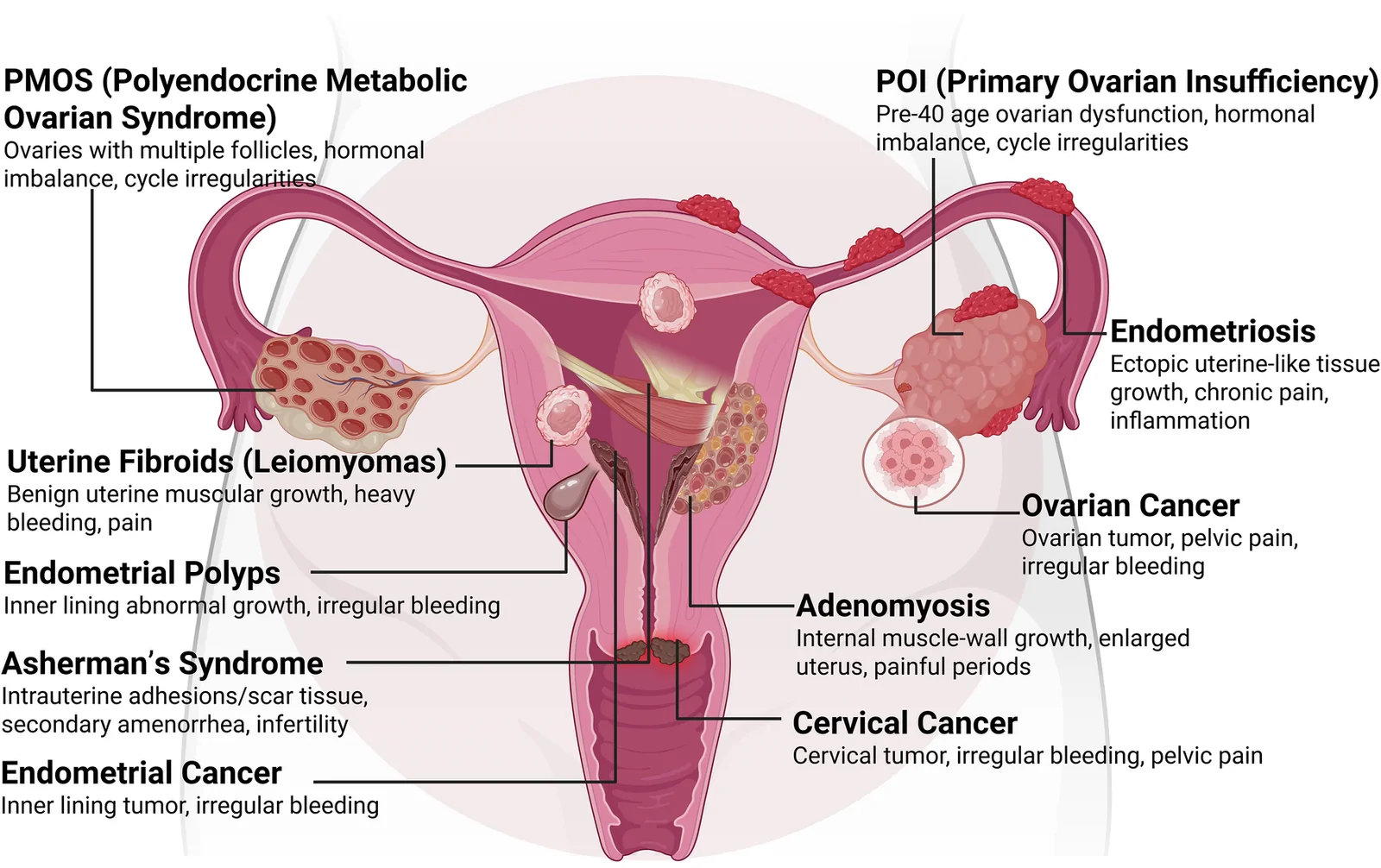
Pathological landscape of gynecological disorders. Anatomical overview and clinical characteristics of major disorders in the female reproductive system. Pathologies are broadly classified by anatomical site and primary clinical manifestation, including ovarian disorders such as Polyendocrine Metabolic Ovarian Syndrome (PMOS) and Primary Ovarian Insufficiency (POI); benign uterine lesions and structural abnormalities such as uterine fibroids (Leiomyomas), endometrial polyps, and adenomyosis; intrauterine scarring characterized by Asherman’s Syndrome; and tissue remodeling or malignant conditions including endometriosis, ovarian cancer, endometrial cancer, and cervical cancer. Brief clinical annotations highlight core pathophysiological features, including hormonal dysregulation, abnormal tissue proliferation, chronic inflammation, irregular uterine bleeding, and associated reproductive impacts. Created in BioRender. Khatun, M. (2026) https://BioRender.com/33l5n8c.

To bridge population-level genetic associations with mechanistic studies of reproductive biology, we propose a PRS-integrated iPSC framework that links inherited genetic susceptibility to functional disease modeling in gynecological disorders (Fig. 1). Individuals are stratified by validated, disease-specific PRS distributions, and somatic cells from selected donors are reprogrammed into iPSCs to establish biobanks. This strategy generates experimentally tractable cohorts spanning the spectrum of polygenic liability, enabling investigation of how cumulative genetic risk influences cellular phenotypes, molecular pathways, and disease-relevant biological processes^9^.

A practical implementation strategy is to prioritize individuals with both a clinically confirmed diagnosis and disease-specific PRS values (e.g., the upper 1–5% of the distribution). Such individuals are expected to carry an enriched burden of genetic susceptibility while also manifesting relevant disease phenotypes, thereby increasing the likelihood of detecting biologically meaningful mechanisms^10^. This enrichment strategy may improve the efficiency of iPSC-based studies by maximizing genetic contrast within experimentally manageable cohorts. Once established, these donor-derived iPSC cohorts can be differentiated into disease-relevant cell types and used to investigate how inherited genetic variation contributes to pathological phenotypes.

The construction of robust and reproducible PRS requires rigorous methodological approaches that account for linkage disequilibrium (LD) structure and effect-size shrinkage, including methods such as LDpred2, PRS-CS, or SbayesR. PRS performance should be evaluated using measures of discrimination (e.g., Area Under the Curve; AUC or C-statistics), calibration, and ancestry-stratified validation to ensure reproducibility and generalizability across populations, as demonstrated in large-scale evaluation studies^11,12^. Although the predictive performance of current PRS for gynecological traits remains modest, particularly in non-European ancestries, their primary role in this framework is not clinical prediction per se^13^. Instead, PRS serves as a research tool for cohort enrichment, enabling the identification of genetically informative donors at the extreme ends of risk distribution. Such extreme-PRS-based selection facilitates the generation of genetically enriched phenotypic groups, an approach that may be particularly valuable for modeling complex disorders such as endometriosis and PMOS in vitro^14^.

## Application to gynecological diseases and malignancies

Endometriosis is a chronic, estrogen-dependent inflammatory disorder affecting approximately 10% of reproductive-age women. Its multifactorial pathogenesis involves profound hormonal dysregulation, immune dysfunction, and genetic susceptibility, with recent studies highlighting substantial pleiotropic genetic overlap between endometriosis and endocrine dysregulation^15^. However, it remains unclear whether inherited risk primarily influences epithelial differentiation, immune dysfunction, stromal remodeling, or a combination of these processes. Within this framework, multi-ancestry PRS-stratified iPSC-derived endometrial epithelial and stromal cells may facilitate investigation of how genetic risk shapes inflammatory signaling, hormonal responsiveness, and lesion-associated phenotypes^14^. When combined with immune cells co-culture and organoid systems, this approach can partially reconstruct the peritoneal inflammatory microenvironment, enabling studies of extracellular matrix remodeling, immune evasion, and localized inflammatory signaling associated with lesion development^16,17^.

PMOS is the most common endocrine disorder, with a prevalence of ~13–20%, and represents a prototypical polygenic condition characterized by hyperandrogenism, ovulatory dysfunction, and metabolic disturbance^18^. Whether genetic susceptibility converges primarily on ovarian dysfunction, systemic metabolic pathways, or both remains incompletely understood. In this context, iPSC-derived ovarian granulosa- and theca-like cells, together with hepatocyte-, adipocyte-, and pancreatic-like lineages, may support multi-tissue modeling of endocrine-metabolic dysregulation^19,20^. Integration with hypothalamic neuron-like cells could further permit partial reconstruction of hypothalamic–pituitary–ovarian (HPO) axis signaling, supporting systems-level investigation of hormone imbalance across distinct polygenic risk backgrounds^21^.

Uterine disorders, including fibroids, adenomyosis, endometrial polyps, and Asherman syndrome, are characterized by aberrant tissue remodeling, fibrosis, and endocrine dysregulation and have increasingly been linked to genetic susceptibility through GWAS and molecular profiling studies^22–27^. PRS-informed iPSC models may enable the study of progesterone-responsive fibroid growth and the excessive extracellular matrix deposition underlying tissue stiffness^28^. In adenomyosis, iPSC-based systems could be used to interrogate disruption of the epithelial–myometrial interface and associated inflammatory signaling cascades^29^. Similarly, patient-specific organ-on-chip models of Asherman syndrome may recapitulate key features of the uterine microenvironment, enabling the study of how genetic background influences aberrant wound healing and persistent intrauterine fibrosis^30,31^.

Beyond uterine pathologies, iPSC-derived granulosa and germ cell-like models offer opportunities to investigate the mechanisms underlying primary ovarian insufficiency (POI). These systems may help elucidate how polygenic susceptibility interacts with pathogenic variants in genes such as *FOXL2*, *NOBOX*, and *BMP15* to promote follicular depletion and ovarian dysfunction^32,33^. In the longer term, genetically informed POI organoids may contribute to the development of personalized hormonal and fertility-preservation strategies.

Gynecological cancers present distinct opportunities for PRS-informed disease modeling because their biological drivers differ substantially across cancer types. For example, endometrial cancer is predominantly associated with hormone-dependent proliferative signaling and dysregulation of the PI3K/AKT pathways. Integration of PRS with clinical covariates, such as body mass index (BMI), may improve biological stratification and facilitate analysis of genotype-dependent endocrine responses^34,35^. In contrast, endometriosis-associated ovarian cancers are thought to arise through chronic inflammatory and oxidative stress pathways. iPSC-derived systems may therefore provide a platform for modeling stepwise malignant transformation through the sequential introduction of recurrent driver mutations, including *CTNNB1*, *ARID1A*, and *PIK3CA*^36,37^. Such models may help distinguish inherited susceptibility from later somatic evolutionary events.

High-grade serous ovarian cancer is characterized by profound genomic instability and defective DNA repair and is believed to originate predominantly from the fallopian tube epithelium. PRS-informed organoid systems combined with targeted perturbation of genes such as *BRCA1* and *TP53* may provide opportunities to investigate early transformation events and the mechanisms governing genomic instability^38,39^. Cervical cancer differs fundamentally from other gynecological malignancies because persistent infection with high-risk human papillomavirus (HPV) is required for tumor initiation^40^. Consequently, a major challenge is understanding how genetic variation shapes host–virus interactions and immune surveillance, not de novo tumor initiation. In this setting, PRS-integrated cervical epithelial and immune co-culture models may offer valuable platforms for dissecting host–virus interactions and identifying determinants of susceptibility to HPV-associated carcinogenesis^41,42^.

## Integration with bioengineering and multi-omics

Because gynecological disorders are profoundly influenced by cyclic hormonal signaling along the HPO axis, reproducing physiological fluctuations in estrogen and progesterone is essential for achieving biological fidelity in in vitro models. Organ-on-chip platforms incorporating microfluidic control systems provide precise and programmable delivery of ovarian hormones to iPSC-derived organoids, allowing the reconstruction of physiologically relevant endocrine states^43,44^. For example, cyclic exposure to estrogen and progesterone can be programmed to mimic follicular- and luteal-phase hormonal profiles, allowing characterization of phase-specific disease phenotypes. Such systems offer a unique opportunity to examine how inherited genetic risk modulates tissue responses to dynamic endocrine cues, a defining feature of disorders such as endometriosis and PMOS^13,45^.

Complementing these bioengineered platforms, single-cell and spatial multi-omics technologies, including single-cell RNA sequencing (scRNA-seq), assay for transposase-accessible chromatin sequencing (ATAC-seq), and spatial transcriptomics, provide high-resolution mapping of cellular states, lineage trajectories, and regulatory networks^46^. When integrated with computational genomics and multimodal machine learning frameworks, these datasets can support the reconstruction of genotype–phenotype relationships and disease trajectories across molecular, cellular, and tissue scales. Collectively, the convergence of advanced bioengineering and multi-omics technologies provides a multidimensional framework for dissecting disease-associated pathways and cellular responses within genetically stratified iPSC models^43,47^.

## Technical, ethical, and translational considerations

### Technical hurdles in iPSC research

Despite major advances in stem cell biology and genome engineering, iPSC-based modeling of polygenic diseases remains constrained by incomplete epigenetic resetting, inter-line variability, and the incomplete maturation of differentiated cell types^48^. These challenges introduce substantial biological and technical variability that can mask the modest effect sizes expected in PRS-stratified systems. Reproducibility is further complicated by differences in differentiation protocols, batch effects, and lineage-specific responses, particularly in endocrine- and immune-responsive cell populations, where physiological fidelity is critical^49^.

Addressing these limitations requires a shift from traditional low-sample experimental designs toward cohort-scale studies capable of capturing the subtle effects of polygenic variation. Such approaches demand rigorous quality control measures, including karyotyping, standard differentiation protocols, next-generation sequencing (NGS)-based evaluation of genomic integrity, and potential off-target assessments. These safeguards are essential to ensure that observed phenotypes reflect underlying biological mechanisms rather than technical artifacts. Consequently, single-line or small cohort studies are generally underpowered to resolve complex polygenic effects revealed by GWAS and PRS analysis^50^.

### The transferability crisis, biobanks, and engineered PRS models

A central paradox in modeling polygenic disease is the mismatch between population-scale genetic discovery and experimentally tractable systems. GWAS typically require hundreds of thousands to millions of individuals (*n* ≈ 10⁵–10⁶) to identify risk loci, whereas iPSC-based studies are generally limited to tens of donors (*n* ≈ 10–100). This disparity restricts the extent to which genome-wide genetic liability can be represented in vitro and underscores that donor-derived iPSC systems function primarily as platforms for functional interrogation rather than direct analogs of clinical PRS^51^.

One practical strategy for generating PRS‑informed iPSC models is through large-scale population biobanks (e.g., Estonian biobank, FinnGen, and UK biobank) that integrate genetic, clinical, and biospecimen data required for reprogramming. Estonian biobank provides a compelling example: with over 20% population coverage^52^, dense SNP‑array genotyping, and validated PRS for major complex diseases, including female reproductive health diagnoses^53^, it enables the identification of individuals at the extremes of genetic predisposition and re‑contact of individuals at the extremes of disease-specific genetic risk under Estonia’s established ethical and legal framework, facilitating the collection of biological samples for generating standardized iPSC lines. These PRS‑stratified iPSC lines, linked to deep longitudinal health records, can then be differentiated into the tissue models most relevant for mechanistic studies of reproductive disease etiologies. More broadly, population-scale biobanks enable efficient identification of genetically informative donors, allowing a relatively small number of iPSC cohorts to capture substantial variation in inherited disease liability while remaining experimentally feasible.

An alternative approach addresses the limited representation of high-risk individuals and the restricted genetic diversity available in small iPSC cohorts. We propose the concept of “isogenic polygenicity”, whereby multiplexed CRISPR/Cas9 editing introduces defined combinations of disease-associated variants into a controlled genetic background, generating engineered polygenic “synthetic PRS” models^54,55^. Unlike natural PRS, which captures the cumulative effects of thousands of variants distributed across the genome^56^, synthetic PRS models incorporate experimentally prioritized subsets of polygenic architecture. Thus, using this approach, isogenic iPSC lines carrying heterozygous or homozygous risk alleles at putative causal variants can then be differentiated into tissue‑specific models. This strategy enables direct investigation of variant interactions, pathway perturbations, and causal relationships that are otherwise difficult to resolve in donor-derived systems^50,54^. Selection of variants for multiplex editing should be guided by convergent genetic and functional evidence, including GWAS significance, fine-mapping posterior probabilities, expression quantitative trait locus (eQTL) associations, chromatin interaction data, and pathway-level relevance. Fine-mapping approaches such as SuSiE or SuSiEx can prioritize likely causal variants, followed by functional annotation-based refinement^57^. To further improve the biological relevance of engineered variants, we recommend employing functional annotation frameworks, such as AlphaGenome^58^, which has demonstrated the ability to predict the functional consequences of non‑coding variants across multiple genome regulatory modalities and to recapitulate known disease‑associated regulatory mechanisms. Therefore, AlphaGenome models can be effectively applied to prioritize non‑coding risk SNPs within GWAS‑derived variant datasets, enabling the selection of the most promising candidates for validation using a “synthetic PRS” model.

**Fig. 2:**
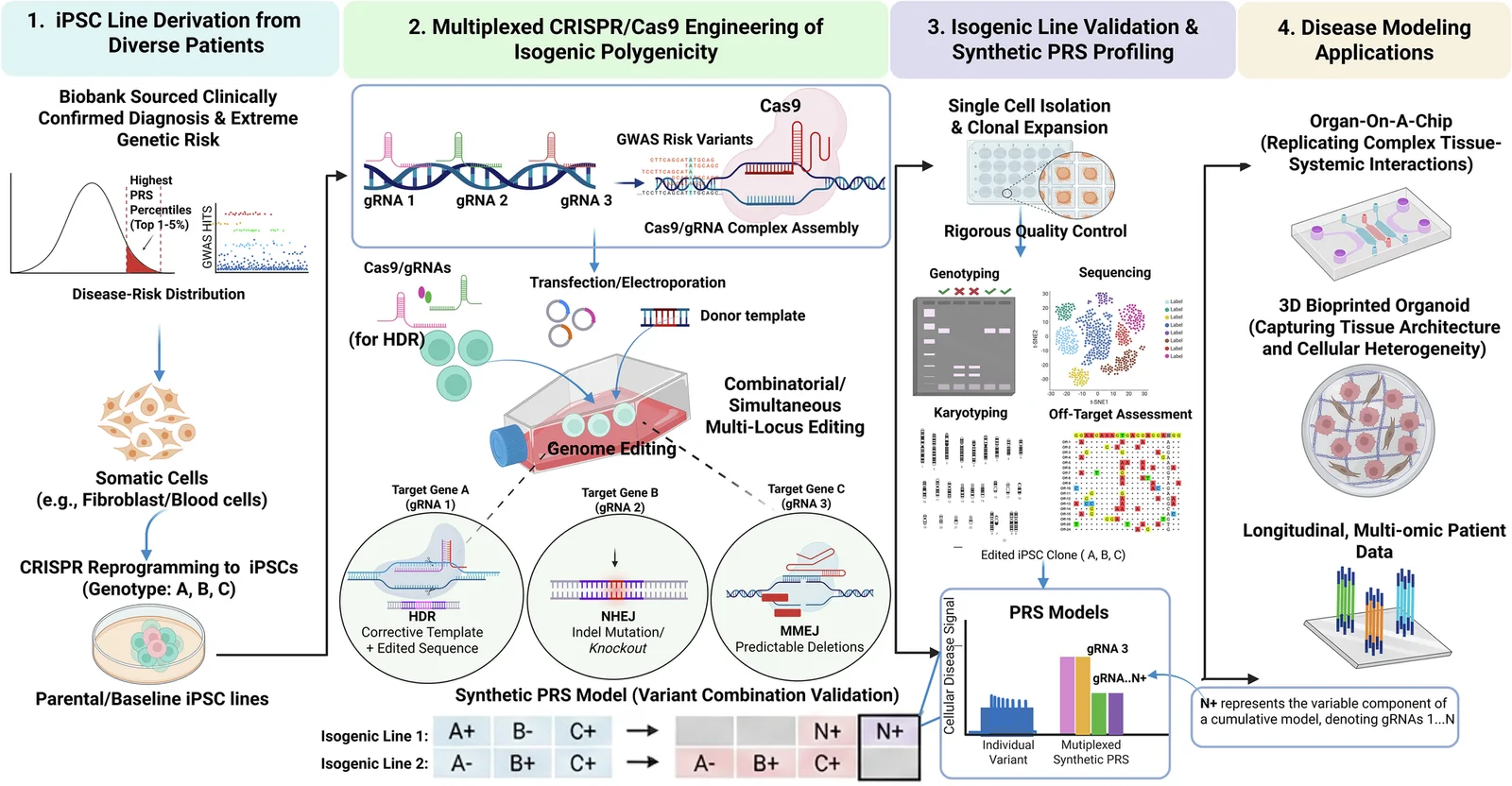
Methodological pipeline for generating synthetic polygenic risk score (PRS) models via multiplexed CRISPR/Cas9 editing. (**1**)** iPSC line derivation**. Somatic cells (e.g., fibroblasts or peripheral blood mononuclear cells) are sourced from biobanks with clinically confirmed diagnoses and extreme genetic risk (top PRS percentiles). These cells are reprogrammed into induced pluripotent stem cells (iPSCs) using transgene-based or non-integrating reprogramming methods by CRISPR/Cas9, thereby preserving the donor´s native genetic background. (**2**)** Multiplexed CRISPR/Cas9 engineering of isogenic polygenicity**. Guide RNAs (gRNA 1- N) are complexed with Cas9 to simultaneously target multiple genome-wide association study (GWAS)-identified variants. Genome editing is achieved through Homology-Directed Repair (HDR) for precise allele introduction/correction, and through Non-Homologous End Joining (NHEJ) or Microhomology-Mediated End Joining (MMEJ) for targeted gene disruption or deletion. This permits the generation of multiple isogenic iPSC lines encoding a defined combination of risk or protective alleles, representing “synthetic PRS” architecture. (**3**) **Isogenic line validation and synthetic PRS profiling**. Following single-cell isolation, clonal lines undergo rigorous quality control, including genotyping, sequencing, karyotyping, and comprehensive off-target assessment using next-generation sequencing. These steps ensure genomic integrity and confirm that engineered variant combinations reflect the intended polygenic architecture. (**4**)** Disease modeling applications**. Validated isogenic lines are differentiated into relevant cell types and used in advanced models, including multi-lineage differentiation platforms, Organ-on-a-Chip systems that recapitulate tissue-systemic interactions, and three-dimensional (3D) bio-printed organoids capturing structural and cellular heterogeneity. These models can be coupled with multi-omic and longitudinal datasets to elucidate epistatic interactions and pathway-level disease phenotypes in a controlled genetic background. Created in BioRender. Khatun, M. (2026) https://BioRender.com/on2l80z.

Within synthetic PRS models, matched risk-enriched and control isogenic lines can be generated through targeted introduction or reversion of prioritized alleles, allowing causal inference by isolating variant-specific effects from broader genetic heterogeneity^59,60^. However, whether multiplex-edited systems faithfully reproduce phenotypes observed in naturally high-PRS individuals remains largely unvalidated. Consequently, donor-derived high-PRS iPSC lines remain essential for preserving endogenous polygenic structure^10^, while multiplexed synthetic PRS models should be viewed as complementary platforms for mechanistic dissection rather than replacements for naturally occurring genetic backgrounds^56,61^. As illustrated in Fig. 2, this proposed workflow integrates donor-derived and engineered approaches, encompassing iPSC derivation, CRISPR-based editing of prioritized variants, clonal expansion, and rigorous quality control procedures, including genotyping, Sanger sequencing, karyotyping, and next-generation sequencing-based assessment of off-target effects.

### Challenges in the GWAS Landscape

A major limitation of current genomic resources is the underrepresentation of diverse ancestral populations in GWAS datasets, which reduces the predictive performance and transferability of PRS across populations. Because PRS accuracy depends on ancestry-specific linkage disequilibrium patterns, allele-frequency distributions, and effect size estimates, models often perform poorly when applied outside the ancestry of the discovery cohort. Consequently, genetic risk may be systematically over- or underestimated in underrepresented groups, potentially reinforcing existing health disparities^5,11^.

These limitations are particularly relevant in gynecological disorders, where disease prevalence, severity, and clinical presentation differ substantially across populations. For example, uterine fibroids are more common and frequently more severe among individuals of African ancestry, while endometriosis remains substantially underdiagnosed in Black populations because of longstanding structural and diagnostic biases within healthcare systems^62^. As a result, inequities in genomic representation can intersect with clinical biases, further complicating disease-risk estimation and interpretation across ancestries.

Improving PRS transferability requires both methodological and data-driven advances. Multi-ancestry PRS approaches, such as PRSmix, can partially address population-specific biases by leveraging genetic information across diverse cohorts, although predictive performance may still vary substantially depending on the ancestry composition of the discovery and target populations^63^. Accordingly, studies should transparently report ancestry-specific performance metrics and clearly communicate the limitations of PRS interpretation across populations.

While iPSC-based disease models provide a valuable platform for testing whether disease-associated pathways are conserved across genetic backgrounds, they cannot overcome biases introduced at the level of GWAS discovery or PRS construction. Addressing these challenges, therefore, requires upstream improvements, including larger-scale multi-ancestry GWAS cohorts, enhanced crossed-population fine-mapping strategies, and explicit quantification of uncertainty in underrepresented groups. Together, these efforts are essential to ensure that PRS-informed experimental systems are not only biologically informative but also representative, equitable, and broadly applicable across diverse populations^5,15^.

### Limitations in oncology and ethics

Although PRS has shown promise for risk stratification in several gynecological malignancies, its predictive performance remains modest and is generally insufficient to replace established clinical risk models^34^. In most settings, PRS provides incremental value when integrated with clinical, environmental, and family-history data rather than functioning as standalone predictors^41^. This limitation reflects the multifactorial nature of cancer susceptibility, which is shaped not only by common genetic variation but also by rare pathogenic variants, epigenetic regulation, environmental exposures, and stochastic biological processes. Accordingly, the primary value of PRS-informed iPSC models currently lies in mechanistic discovery, pathway interrogation, and functional validation of disease-associated genes and networks rather than direct clinical prediction^64^.

Another major limitation is that PRS do not capture rare, high-penetrance pathogenic variants (e.g., in *BRCA1/2*, *PTEN*, or mismatch repair genes), which can independently confer substantial cancer susceptibility. To avoid conflating monogenic and polygenic risk, PRS-based studies should be complemented by whole-exome or whole-genome sequencing. This approach enables carriers of high-risk variants to be analyzed separately and facilitates independent evaluation of monogenic and polygenic disease mechanisms^65^.

Beyond these scientific considerations, PRS-integrated iPSC cancer research raises critical ethical and governance challenges. These include the interpretation and disclosure of genetic risk information, management of incidental findings, potential psychological impacts on participants, and long-term stewardship of genomic and biobank data. Addressing these issues requires robust informed consent processes, access to genetic counseling, secure data-governance frameworks, and coordinated ethical oversight, particularly given the limited regulatory guidance currently available for PRS-integrated iPSC research. Effective implementation will also depend on transparent communication, participant education, and evidence-based counseling strategies^66,67^.

## Conclusion, proposed solutions, and future directions

**Fig. 3:**
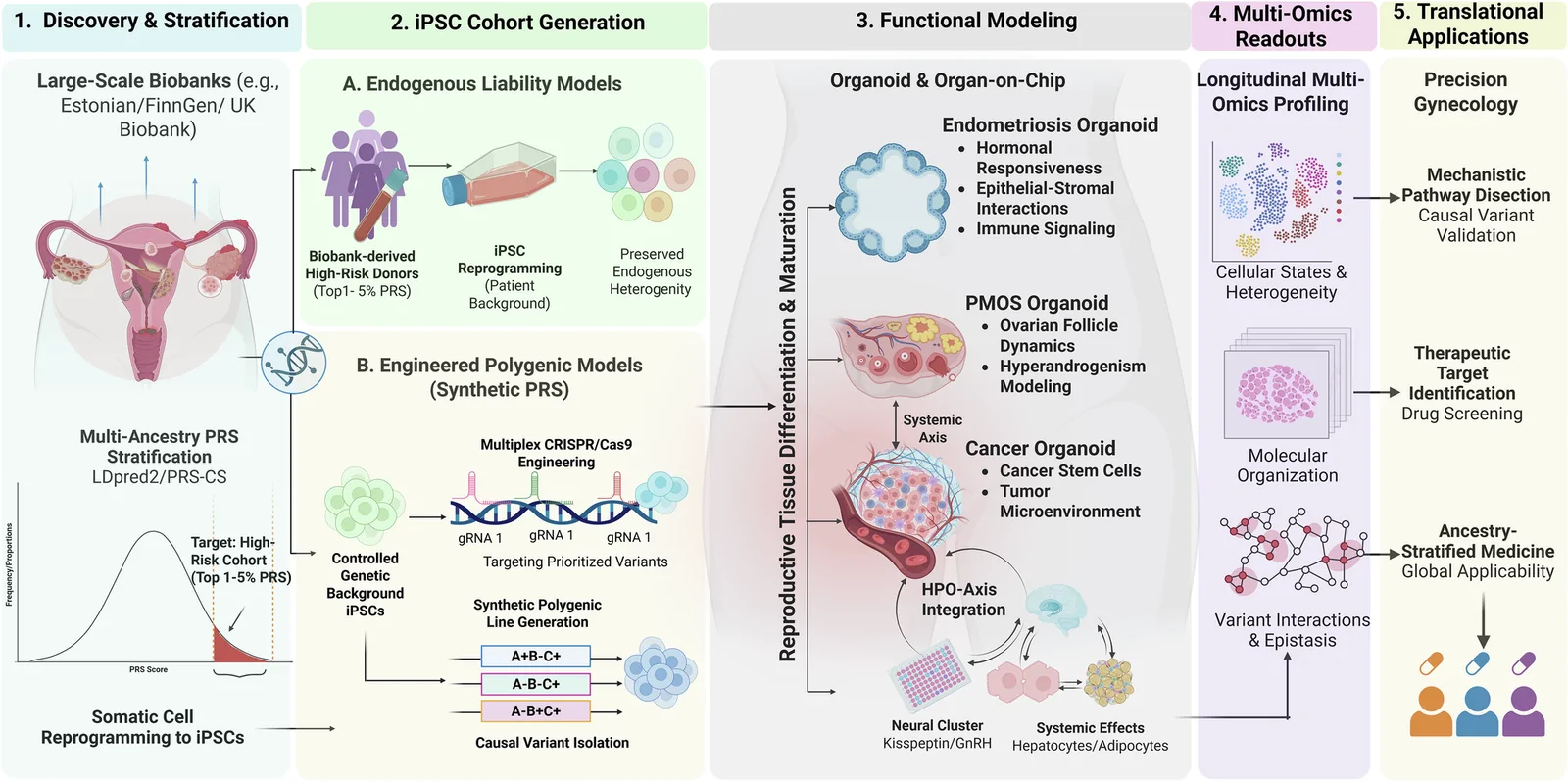
PRS-iPSC conceptual framework. This framework outlines an integrative strategy to investigate the functional consequences of inherited genetic variation in complex gynecological disorders. (**1**)** Discovery and stratification**. Large-scale population biobanks, such as Estonian, FinnGen, and UK Biobank, are used to identify individuals with clinically confirmed gynecological conditions. Donors are stratified using validated multi-ancestry polygenic risk scores (PRS), constructed with methods such as LDpred2 or PRS-CS, to enrich individuals in the upper percentiles of genetic liability (e.g., top 1–5%). (**2**)** Model generation**. Two complementary strategies can be employed: (**A**) **Endogenous liability models**, where somatic cells from high-PRS individuals are reprogrammed into iPSCs, preserving the native polygenic background. (**B**) **Engineered polygenic models (synthetic PRS)**, where multiplexed CRISPR/Cas9 editing introduces or corrects prioritized risk variants within a controlled genetic background to isolate causal effects. (**3**)** Functional modeling**. iPSC-derived lines are differentiated into disease-relevant cell types and organoid systems modeling key gynecological conditions, including endometriosis, polyendocrine metabolic ovarian syndrome (PMOS), and gynecological cancers. These models are further extended using the hormone–pituitary–ovarian axis (HPO axis)–relevant multi-tissue systems incorporating neural, hepatic, and adipose components to better recapitulate systemic reproductive physiology. (**4**)** Multi-omics readouts**. Longitudinal, high-resolution molecular profiling (e.g., scRNA-seq, ATAC-seq, spatial transcriptomics) is applied to characterize cellular states, regulatory architecture, and variant-level epistatic interactions across disease-relevant contexts. (**5**)** Translational applications**. Integration of these functional and multi-omics data facilitates the mechanistic dissection of disease pathways, validation of causal variants, and clinical-grade drug screening. Together, ancestry-stratified PRS and iPSC-based models provide a scalable framework for advancing equitable, mechanism-driven Precision gynecology. Created in BioRender. Khatun, M. (2026) https://BioRender.com/hdzdi4k.

We propose a conceptual framework that combines PRS-guided donor stratification, iPSC-based disease modeling, multiplex genome engineering, and advanced bioengineering platforms to investigate the functional consequences of inherited genetic variation in gynecological disorders. By integrating naturally occurring polygenic backgrounds with experimentally controlled genetic perturbations, this framework offers complementary approaches for interrogating disease-associated pathways across reproductive tissues (Fig. 3).

Several challenges must be addressed before widespread implementation. These include improving causal variant resolution, enhancing representation of diverse ancestral populations in genomic discovery efforts, enhancing reproducibility across iPSC-derived systems, and more accurately recapitulating endocrine, immune, and tissue microenvironments. Overcoming these limitations will require coordinated advances in statistical genetics, stem cell biology, bioengineering, and computational genomics.

At present, PRS-informed iPSC and organoid systems should be viewed primarily as platforms for mechanistic discovery, target prioritization, and functional validation rather than direct clinical application. Future translational progress will depend on the establishment of robust reproducibility standards, predictive benchmarks, and regulatory frameworks aligned with agencies such as the Food and Drug Administration (FDA), European Medicines Agency (EMA), and Therapeutic Goods Administration (TGA). Continued integration of human genetics, genome engineering, and multi-omic profiling will further strengthen the ability to connect genetic risk with biological function.

Ultimately, the primary value of PRS-informed iPSC systems may lie less in disease risk prediction and more in translating statistical genetic associations into experimentally testable biological mechanisms. By integrating population-scale biobanks, PRS-guided donor stratification, and iPSC-based functional models, this framework provides a practical bridge between population genetics and functional genomics. Complemented by engineered “synthetic PRS” models, it enables systematic dissection of the molecular and cellular pathways underlying complex gynecological diseases. Collectively, these approaches establish a foundation for mechanism-driven precision gynecology, linking genetic risk to disease biology, therapeutic targets, and future intervention strategies.
